# The Advantage of Synthetic MRI for the Visualization of Early White Matter Change in an Infant with Sturge-Weber Syndrome

**DOI:** 10.2463/mrms.ci.2015-0164

**Published:** 2016-03-21

**Authors:** Christina ANDICA, Akifumi HAGIWARA, Misaki NAKAZAWA, Kouhei TSURUTA, Nao TAKANO, Masaaki HORI, Hiroharu SUZUKI, Hidenori SUGANO, Hajime ARAI, Shigeki AOKI

**Affiliations:** 1 Department of Radiology, Juntendo University School of Medicine, 2-1-1 Hongo, Bunkyo-ku, Tokyo 113-8421, Japan; 2 Department of Radiology, The University of Tokyo; 3 Department of Radiological Sciences, Tokyo Metropolitan University; 4 Department of Neurosurgery, Juntendo University School of Medicine

**Keywords:** synthetic MRI, magnetic resonance quantification, Sturge-Weber syndrome, accelerated myelination

Sturge-Weber syndrome (SWS) is a developmental disorder with leptomeningeal angiomatosis as a major pathological abnormality.^1^ In an infant with SWS, the white matter underlying the angiomatosis typically shows prominent hypointensity on T_2_-weighted image (WI) compared to the remainder of the brain. This hypointensity can be caused by several factors, and one possible explanation for this finding is increased myelination or some authors call it “accelerated myelination.”^1,2^

Quantification magnetic resonance imaging (MRI) is a technique to quantify the longitudinal T_1_ relaxation, the transverse T_2_ relaxation, the proton density (PD), and the amplitude of the local radio frequency B_1_ field. Quantification performed by QRAPMASTER (Quantification of Relaxation Times and Proton Density by Multiecho Acquisition of a Saturation-recovery using Turbo spin-Echo Readout) pulse sequence with multi-slice, multi-echo, and multi delay acquisition.^3^ Based on these data, any contrast-weighted images with the combination of echo time (TE), repetition time (TR), and inversion time (TI) can be created and contrast-weighting can be freely adjusted retrospectively.

A 4-month-old male infant was referred to our hospital with a few episodes of left leg twitching. Clinical examination showed a right facial angiomatosis and a left leg hemiparesis. A 3.0T MR system (Discovery MR750w, GE Healthcare, Milwaukee, USA) with a 12-channel head coil was used for conventional and synthetic imaging. Synthetic images were created using SyMRI StandAlone software (SyntheticMR AB, Linköping, Sweden). It takes 7 minutes 12 seconds for quantification.

Contrast enhanced (CE) conventional brain MRI showed right cerebral hemisphere atrophy and ipsilateral leptomeningeal angiomatosis. Synthetic MRI can show the “accelerated myelination” more clearly. In infants less than 12 months old, heavily T_2_-weighted sequences are highly recommended, as the water content of the brain in younger children is considerably higher than in older children and adults.^1^ It is also reported that myelination process is associated with T_1_ and T_2_ shortening, and also with decreasing PD.^1^ Synthetic T_2_WI with longer TR and TE showed abnormal white matter hypointensity on the “accelerated myelination” area (Fig. 1a TR 15,000 ms; TE 200 ms), better than conventional T_2_WI (Fig. 1b TR 4500 ms; TE 111.36 ms). The quantitative map (Fig. 1c) showed that all the T_1_, T_2_, and PD values of the “accelerated myelination” areas (1104 ms, 91 ms, 77.1 percentage unit [pu]) were lower than the contralateral area (1239 ms, 108 ms, 81.1 pu).

Double inversion-recovery (DIR) was used in non-CE and CE synthetic MR images. In non-CE synthetic image, DIR was used to suppress the non-myelinated area and cerebrospinal fluid (CSF) so that the myelinated area could be highlighted as hyperintensity (Fig. 1d TR 15,000 ms; TE 100 ms, 1st TI 990 ms, 2nd TI 4920 ms). CE synthetic DIR clearly demonstrated leptomeningeal angiomatosis by nulling the CSF and minimizes the signal of fat in the bone marrow and subcutaneous tissue.^4^

Synthetic T_2_WI with longer TR and TE and DIR images is useful to visualize early white matter change in an infant with SWS. This white matter change, so called “accelerated myelination” is one of the early signs of SWS and detection of this finding may improve the prognosis by preventive anti-epileptic treatment.^2^

## Figures and Tables

**Fig. 1.**
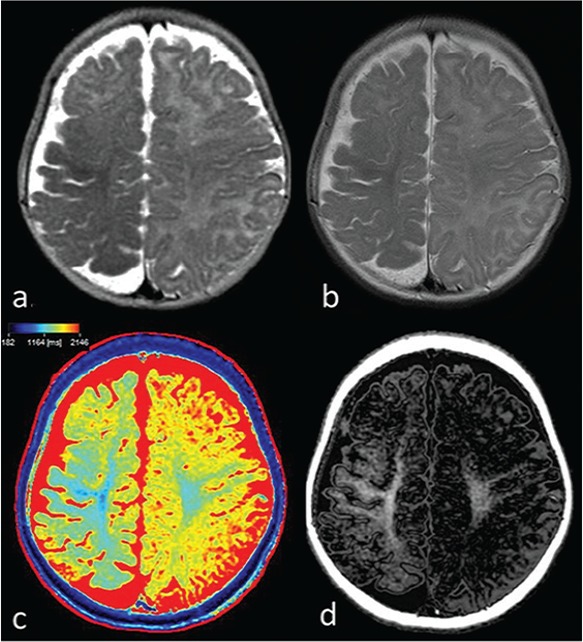
A 4-month-old male infant with Sturge-Weber syndrome. Synthetic T_2_WI with longer repetition time and echo time (**a**) shows abnormal white matter hypointensity better than conventional T_2_WI (**b**). The quantitative map (only T_1_ map is shown here) (**c**) shows that T_1_, T_2_, and proton density values on the “accelerated myelination” area are decreased. Synthetic double inversion-recovery image (**d**) which suppresses the signals of non-myelinated white matter and CSF is used for highlighting the increased myelination area. WI, weighted image.
